# Dynamic tumor modeling of the dose–response relationship for everolimus in metastatic renal cell carcinoma using data from the phase 3 RECORD-1 trial

**DOI:** 10.1186/1471-2407-12-311

**Published:** 2012-07-23

**Authors:** Andrew Stein, Wenping Wang, Alison A Carter, Ovidiu Chiparus, Norbert Hollaender, Hyewon Kim, Robert J Motzer, Celine Sarr

**Affiliations:** 1 Modeling & Simulation, Novartis Institutes for Biomedical Research, 45 Sidney St, Cambridge, MA, USA; 2 Novartis Pharmaceuticals Corporation, East Hanover, NJ, USA; 3 Novartis Pharmaceuticals Corporation, East Hanover, NJ, USA; 4 Novartis Pharmaceuticals Corporation, East Hanover, NJ, USA; 5 Novartis Pharma AG, Basel, Switzerland; 6 Department of Experimental and Clinical Pharmacology, University of Minnesota, Minneapolis, MN, USA; 7 Department of Medicine, Genitourinary Oncology Service, Memorial Sloan-Kettering Cancer Center, New York, NY, USA; 8 Novartis Pharmaceuticals Corporation, East Hanover, NJ, USA

**Keywords:** Efficacy, Kidney, Oncology, Pharmacodynamics, Therapeutics

## Abstract

**Background:**

The phase 3 RECORD-1 trial (NCT00410124) established the efficacy and safety of everolimus in patients with metastatic renal cell carcinoma (mRCC) who progress on sunitinib or sorafenib. In RECORD-1, patients received 10 mg everolimus daily, with dose reduction to 5 mg daily allowed for toxicity. We have developed a model of tumor growth dynamics utilizing serial measurements of the sum of the longest tumor diameters (SLD) from individual RECORD-1 patients to define the dose–response relationship of everolimus.

**Results:**

The model predicts that after 1 year of continuous dosing, the change in SLD of target lesions will be +142.1% ± 98.3%, +22.4% ± 17.2%, and –15.7% ± 11.5% in the average patient treated with placebo, 5 mg everolimus, and 10 mg everolimus, respectively. This nonlinear, mixed-effects modeling approach can be used to describe the dynamics of each individual patient, as well as the overall population. This allows evaluation of how an actual dosing history and individual covariates impact on the observed drug effect, and offers the possibility of predicting clinical observations as a function of time.

**Conclusions:**

In this pharmacodynamic model of tumor response, everolimus more effectively shrinks target lesions in mRCC when dosed 10 mg daily versus 5 mg daily, although a 5-mg dose still shows an antitumor effect. These data support earlier studies that established 10 mg daily as the preferred clinical dose of everolimus, and improve our understanding of the everolimus dose–response relationship.

## Background

Everolimus is an orally active inhibitor of the mammalian target of rapamycin (mTOR) that has been approved in 65 countries worldwide for the treatment of patients with metastatic renal cell carcinoma (mRCC) who progress on or are intolerant of vascular endothelial growth factor receptor-tyrosine kinase inhibitor (VEGFr-TKI) therapy [1].

The serine/threonine kinase mTOR is a key regulator of cell growth and proliferation, metabolism, and angiogenesis [2]. Abnormal activation of the mTOR signaling pathway has been implicated in the pathogenesis of RCC [3-5]. Two principal downstream effectors are responsible for relaying mTOR signaling: the translational repressor protein eukaryotic initiation factor 4E (eIF-4E) binding protein 1 (4EBP1) and the ribosomal protein S6 kinase 1 (S6K1) [6]. Phosphorylation of 4EBP1 by mTOR causes the release eIF-4E, which then acts to initiate cap-dependent protein translation. Following activation by mTOR, S6K1 also regulates protein translation via phosphorylation of ribosomal protein S6.

Results of preclinical and clinical studies have demonstrated the relationship between inhibition of mTOR signaling by everolimus and antitumor efficacy [7-10]. Doses of everolimus that produced an antitumor effect in a syngeneic CA20948 pancreatic rat tumor xenograft model also dramatically inhibited mTOR signaling (as measured by inhibition of 4E-BP1 phosphorylation and S6K1 signaling) in tumor, skin, and peripheral blood mononuclear cells (PBMCs) [10]. These data were used to develop a direct-link pharmacokinetic/pharmacodynamic model that described the relationship between inhibition of S6K1 and antitumor effects of different concentrations of everolimus in tumor-bearing rats. Once corrected for interspecies pharmacokinetic differences, this model was applied in a phase 1 dose-escalation trial to describe changes in S6K1 inhibition in tumor and PBMCs from patients treated with everolimus [11]. The model predicted that daily doses of everolimus 5 or 10 mg/day would demonstrate a more profound and sustained effect on S6K1 inhibition than weekly doses of 20, 30, 50, or 70 mg. A subsequent phase 1 dose-escalation study evaluated the pharmacodynamic effects of the above doses and schedules of everolimus using biomarkers from both the 4E-BP1 and S6K1 pathways [11]. Inhibition of mTOR was achieved at all doses and schedules; however, more profound inhibition of the pathway was seen with 10 mg daily than with 5 mg daily or any weekly dosing schedule, as this was the only dose that achieved complete inhibition of both the 4E-BP1 and S6K1 pathways. Based on these phase 1 data, a daily dose of 10 mg of everolimus was used in a subsequent phase 2 study in patients with mRCC [12], and in the pivotal phase 3 RECORD-1 trial [13,14].

The RECORD-1 trial was an international, randomized, placebo-controlled study that demonstrated the efficacy and safety of everolimus over placebo in patients with mRCC who progressed after initial treatment with VEGFr-TKIs [13,14]. The primary efficacy end point for the study was progression-free survival (PFS) per central radiology review, according to RECIST [15]. Median PFS was more than doubled by everolimus (4.9 months) versus placebo (1.9 months) (HR, 0.33; 95% CI: 0.25-0.43; *P* < 0.001), thus establishing the efficacy of everolimus in patients with mRCC [13,14]. Everolimus was also more effective than placebo at reducing tumor size; 47% of everolimus-treated patients showed a decrease in the sum of the longest tumor diameters (SLD), compared with 10% of patients receiving placebo [14]. Notably, a retrospective analysis of RECORD-1 identified a best overall tumor burden reduction of 5% as the threshold for PFS benefit in everolimus-treated patients [16].

The RECIST criteria divide patients into 4 response categories: complete response (CR), partial response (PR), stable disease (SD), and progressive disease (PD), based on changes in the SLD of target lesions, the unequivocal progression or disappearance of nontarget lesions, and the appearance of new metastases. While RECIST provides a categoric assessment of patient response, the change in an individual patient’s tumor size over time provides a continuous assessment of response. As such, monitoring change in tumor size over time may enable detection of therapeutic efficacy using a smaller number of patients than required with RECIST [17]. Pharmacodynamic models of the change in tumor size over time have been used recently to quantify the effects of therapy on solid tumors [18,19]. Such models may provide information about a variety of factors affecting patient response to a drug, including demographics, stage of disease, or baseline biomarkers, and are expected to have an enhanced ability to detect the prognostic significance of such factors as compared with analyses that employ time-to-event data such as PFS [20]. The use of dynamic tumor models to link change in tumor size over time to patient response and survival has been proposed as a tool for improving clinical trial design and decision-making in oncology drug development [20,21].

Herein, we report our development of a model for tumor growth dynamics to describe the tumor burden reduction response to everolimus in the phase 3 RECORD-1 trial in patients with mRCC. This model was used to explore the effect of two different doses of everolimus (5 mg and 10 mg daily) on tumor growth. This methodology complements prior phase 1 analyses of mTOR pathway inhibition used to guide dose selection, and directly links the administered dose of everolimus to change in SLD, a key variable used in the assessment of PFS, the primary end point for the RECORD-1 study.

## Results

Of the 416 evaluable patients from the RECORD-1 trial, 407 (97.8%) had at least 1 baseline tumor assessment by the local investigator and were included in this analysis (Table 1); there were a total of 1569 individual tumor measurements. Baseline tumor measurements were obtained for 272/277 patients in the everolimus arm and 135/139 patients in the placebo arm. The majority (79.9%) of patients on the placebo arm crossed over to everolimus after disease progression, after a mean placebo treatment duration of 96.5 days. A total of 98 patients, 69 patients from the everolimus arm and 29 patients from the placebo arm, received at least 1 dose adjustment of everolimus to 5 mg daily over the course of the trial. Among these 98 patients, the mean duration of everolimus treatment was 117.3 days at 5 mg, and 40.5 days at 10 mg. Sixty-eight of these patients had at least 1 tumor assessment after receiving a 5-mg dose (53 from the everolimus arm and 15 from the placebo arm).

**Table 1:** Regimen and pumor assessment in patients enrolled in the RECORD-1 Phase 3 Trial

**Patient category**	**Total**	**Everolimus arm**	**Placebo arm**
All	416	277	139
With baseline tumor measurement	407	272	135
Crossed over from placebo to everolimus	111	NA	111
Received at least one 5-mg dose of everolimus	98	69	29
Received at least one 5-mg dose and a subsequent tumor size assessment	68	53	15

NA, not applicable.

**Figure 1:**
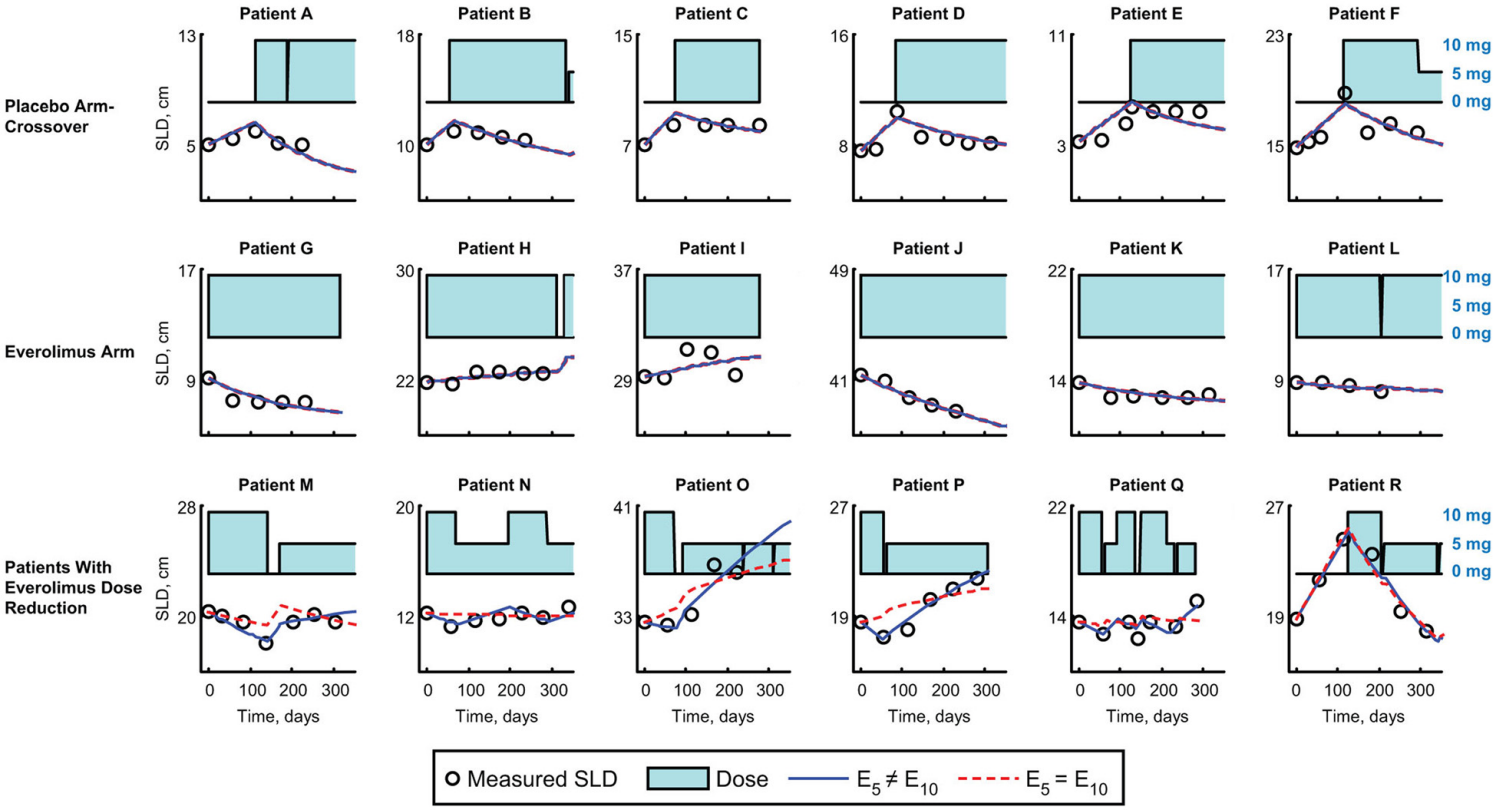
**Change in sum of the longest tumor diameters (SLD) over time in representative patients in relation to everolimus treatment.** Three treatment groups are shown: 1) patients randomized to placebo who crossed over to everolimus on disease progression; 2) patients randomized to everolimus; and 3) patients randomized to everolimus or placebo who had at least one everolimus dose reduction from 10 mg to 5 mg daily. Two different fits of the SLD over time are illustrated for each patient, based on: i) a model that assumes *E*_5_ = *E*_10_ (model 1, shown in the red dashed line) and ii) a model that assumes *E*_5_ ≠ *E*_10_ (model 2, shown in the blue solid line). Daily dose of everolimus administered is indicated by a barplot as a function of time with a value of 10 mg at its maximum. *E*_*5*_, treatment effect of everolimus 5 mg daily; *E*_*10*_, treatment effect of everolimus 10 mg daily.

Representative plots of the measured change in SLD in individual patients over time as related to dose of everolimus received are shown in Figure 1. Data for all 407 patients were fit to 2 nested dose-effect models based on the following equation: *dy/dt = r − E*_*dose*_*y*, where *y* is the SLD, *dy/dt* is the change in SLD, *r* is the placebo growth rate, and *E*_*dose*_ is the tumor shrinkage due to the daily dose, which can change with time. The nesting arises from the use of two different models for *E*_*dose*_ (model 1: 5 mg and 10 mg everolimus have equal effects [*E*_*5*_ *= E*_*10*_]; model 2: 5 mg and 10 mg everolimus have different effects [*E*_*5*_ *≠ E*_*10*_]. It is important to note that the dose of everolimus could change at any given time. Some patients who were randomly assigned to placebo switched to everolimus upon disease progression, and some patients who received everolimus could have had their dose reduced to either 5 mg or 0 mg to manage an adverse event (AE). This modeling approach was designed to account for daily changes in everolimus dose (see the Methods section for a more detailed description of each model and model parameters). Patients on either the placebo arm or the everolimus arm who did not have everolimus dose reduction to 5 mg were well described by both models. However, a clear distinction between the 2 models was evident in the subgroup of patients who had everolimus dose reduction to 5 mg. Model 2, which allowed the effects of 5 mg and 10 mg everolimus to differ, provided a significantly better fit for the data from this patient group than did the model describing an identical effect for the 2 doses (model 1). The improvement of model 2 over model 1 as applied to the entire patient population was quantified using a likelihood ratio test and was found to be highly statistically significant (*P* < 0.0001). Of the 407 patients evaluated, only 7 patients presented poor fits to the model; of these, 4 patients had an SLD that decreased during initial treatment with everolimus, but began to increase again after prolonged treatment ( Additional file 1: Figure A1).

**Figure 2:**
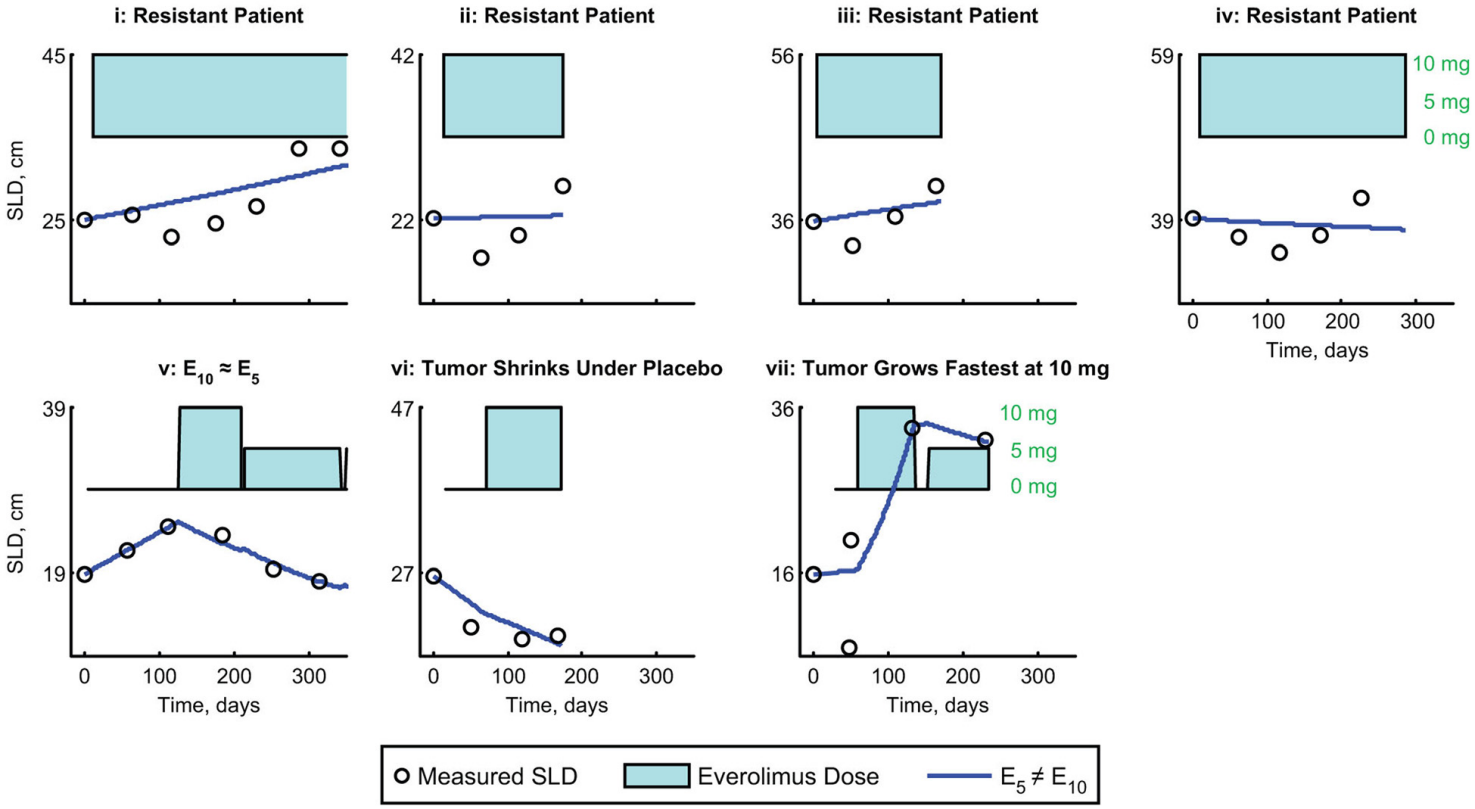
**Tumor growth in a small number of RECORD-1 patients was not well described by model 2 with E**_**5**_ **≠ E**_**10**_**.** These included: i-iv) resistant patients (*n* = 4); (v) 5 mg of everolimus had an equivalent effect to 10 mg of everolimus (*n* = 1); (vi) tumor shrinkage during placebo treatment, but progression due to nontarget lesions (*n* = 1); (vii) tumor growth at 10 mg everolimus is faster than tumor growth at 5 mg everolimus (*n* = 1). E_5_, treatment effect of everolimus 5 mg daily; E_10_, treatment effect of everolimus 10 mg daily; SLD, sum of the longest tumor diameters.

Parameters for the typical patient in model 2 were, *r* = 46.0 ± 5.7 10^-3^ cm/day, *E*_10_ = 3.9 ± 0.5 10^-3^/day, and *E*_5_ = 2.3 ± 0.5 10^-3^/day. The η-shrinkage on each parameter was η_r_ = 0.21, η_E10_ = 0.36, and η_E5_ = 0.80. The large η shrinkage for E_5_ may arise because this parameter cannot be estimated for many patients who don’t receive a 5-mg everolimus dose. Model simulation, as described in the Methods section, was performed to explore the response of a typical patient maintaining continuous, uninterrupted dosing. The model predicts a change in SLD of target lesions after 3 months of 35.6% ± 24.8% on placebo, +7.7% ± 7.0% for a 5-mg dose, and –7.6% ± 5.5% for a 10-mg dose and after 1 year of +142.1% ± 98.3% on placebo, +22.4% ± 17.2% for a 5-mg dose, and –15.7% ± 11.5% for a 10-mg dose (Figure 2).

A covariate analysis was conducted to investigate the impact of prognostic factors of reported relevance in mRCC on the placebo growth rate (*r*) and drug effect (*E*_*10*_, *E*_*5*_). Results of this analysis showed that the only significant predictor of the model parameters was the tumor size at baseline. A larger baseline SLD was indicative of increased placebo growth rate and decreased drug effectiveness with high statistical significance (*P* < 0.0001). Other prognostic factors did not show any significant impact on these parameters. We note that the next most predictive covariate was the corrected calcium levels; however, while this covariate did lead to a numerically better fit, it did not reduce the intersubject variability of the model parameters (η_r_ η_E10_) and thus was not included in the final model.

**Figure 3:**
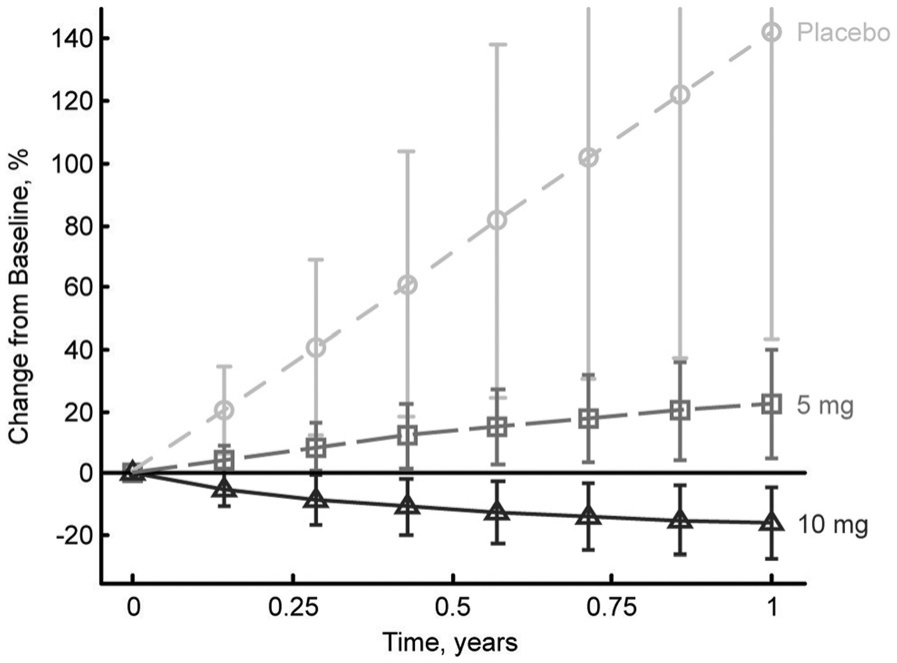
Simulated changes in tumor size over 1 year in the average patient treated continuously with either placebo or everolimus (5 or 10 mg daily).

**Figure 4:**
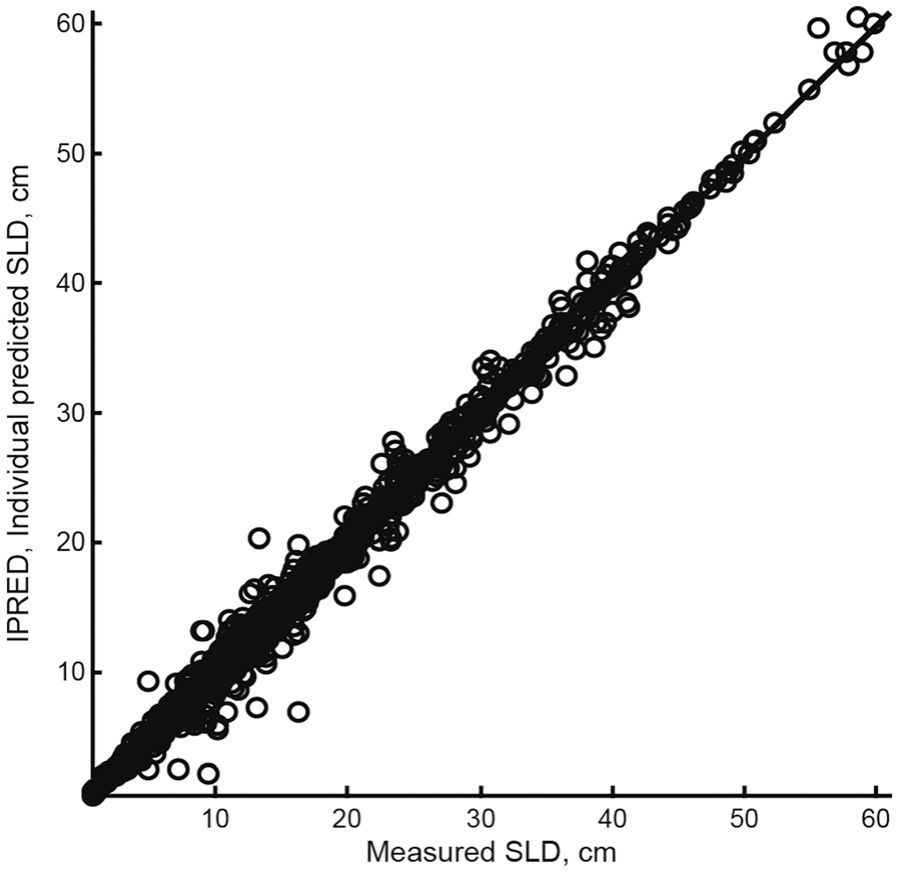
**Correlation of the individual predictions of SLD by the model in which *E* **_**5**_ **≠** ** *E* **_**10 **_**(model 2) to the actual measured SLD from each patient.** E_5_, treatment effect of everolimus 5 mg daily; E_10_, treatment effect of everolimus 10 mg daily; SLD, sum of the longest tumor diameters.

The final model for the *i*th patient is given by

(1) $$\begin{array}{l} dy_{i}/dt=r_{i}-E_{\mathrm{dose}}y_{i}\\ E_{\mathrm{dose}}=\left\{ \begin{array}{c} E_{10,i}\text{ when dose }=10\;\text{mg}\\ E_{5,i}\text{ when dose}=5\;\text{mg}\\ 0\text{ when dose}=0\; \end{array}\right.\\ r_{i}=r\times {\left(y_{0,i}/{\hat{y}}_{0}\right)}^{\theta 1}+N(0,\eta_{r})\\ E_{10,i}=E_{10}\times {\left(y_{0,i}/{\hat{y}}_{0}\right)}^{\theta 2}+N(0,\eta_{E10})\\ E_{5,i}=E_{5}\times {\left(y_{0,i}/{\hat{y}}_{0}\right)}^{\theta 2}+N(0,\eta_{E5})\\ \epsilon =N(0,\sigma_{\epsilon }) \end{array}$$

where *ŷ*_0_ is the median baseline SLD of 14.4 cm, and *N*(0,*η*) indicates a random variable following a normal distribution with mean 0 and standard deviation to be estimated. The model parameters are summarized in Table 2. Model qualification showed: 1) good agreement between the individual, clinically measured values for SLD and those predicted by the model (Figure 3); 2) acceptable uncertainty in parameter values, as measured by the standard error (Table 2); 3) good agreement with actual patient observations in a simulation of two sets of 10,000 virtual patients (Figure 4). The purpose of the visual predictive check (VPC) is to ensure that the model adequately describes the data used to develop it. We note that at early times, there is good agreement between the model simulations and the actual data of the 5th, 50th, and 95th percentiles. However, at later times, the 95th percentile tends to be underestimated. This is due to informative censoring of the trial; patients with fast growing tumors are more likely to progress and then change therapies. For example, a patient on placebo with a rapidly growing tumor may progress after only two months and change therapy, whereas a patient with a slower growing tumor could stay on placebo for 12 months. A more complete simulation of the trial would ultimately require simulation of the effect of tumor growth on the therapy received.

**Table 2:** **Parameters for final model (Model 2 [E**_**5**_ **≠ E**_**10**_**]) with all covariates**

**Variable**	**Units**	**Mean value, *θ* ** **± SE**	**Intersubject variability, *η* ** **± SE**
Placebo growth rate, *r*	10^-3^ cm/day	46.0 ± 5.7	3.5 ± 2.1
Drug effect of 10 mg, *E*_10_	10^-3^/day	3.9 ± 0.5	0.3 ± 0.2
Drug effect of 5 mg, *E*_5_	10^-3^/day	2.3 ± 0.5	0.2 ± 0.2
Multiplicative effect of baseline SLD on placebo growth rate, *θ1*		0.4 ± 0.2	NA
Multiplicative effect of baseline SLD on drug effect, *θ2*		−0.7 ± 0.2	NA
Residual error, σ_ε_	cm	1.1	NA

NA, not applicable; SE, standard error; SLD, sum of the longest tumor diameters.

Intersubject variability (η) denotes the standard deviation of the parameter distributions.

**Figure 5:**
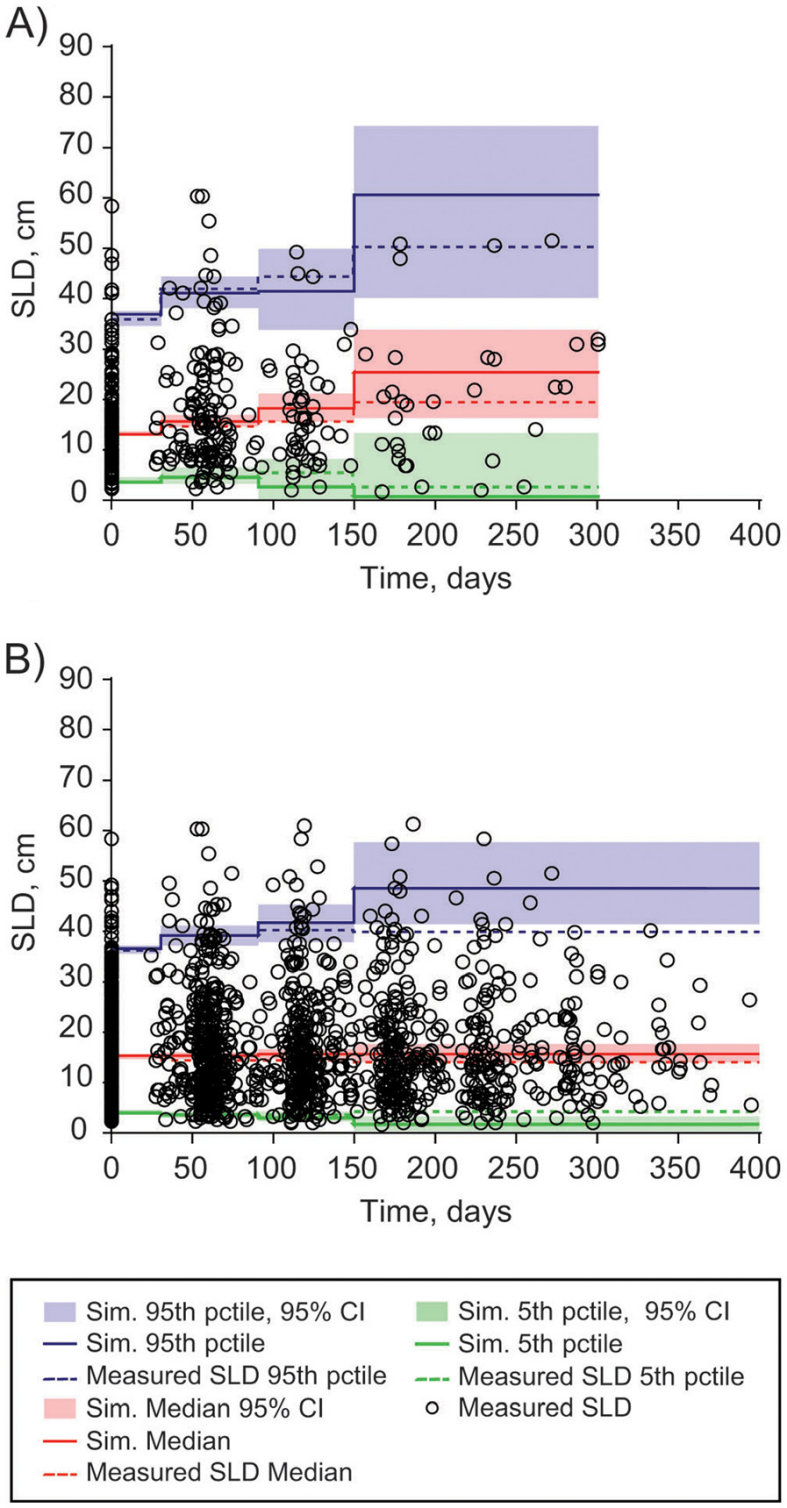
**Visual predictive check (VPC) of 10,000 simulated patients (A) on placebo before their switch to everolimus, and (B) on everolimus who received continuous 10 mg dosing throughout the trial.** CI, confidence interval; pctile, percentile; Sim, simulated; SLD, sum of the longest tumor diameters.

As the trial protocol called for dose reductions only when patients experienced AEs, there is a risk that the dose–response relationship we have observed for 5 mg everolimus only applies to patients who experienced AEs, and not to the entire population. To evaluate this possibility, individual estimates of *E*_*10*_ were compared for the subgroups of patients who did have, or who did not have, any everolimus dose reductions or interruptions. The median drug effect (*E*_*10*_) for these two populations was found to differ by only 4% (Figure 5). The minimal difference in *E*_*10*_ observed between patients who did or did not experience dose reductions or interruptions suggests that conclusions derived from the model can be generalized to the overall population.

## Discussion

We have developed a pharmacodynamic model to describe tumor growth in patients with mRCC enrolled in the phase 3 RECORD-1 trial. Although RECORD-1 did not have a separate arm in which all patients were administered a 5-mg dose of everolimus, the model was able to detect a difference in the effect of a 5-mg and a 10-mg dose on reducing the size of target lesions by taking into account the dosing histories of individual patients (Figure 1). It is worthy of note that when formulating the model, the effect of 5 mg everolimus was permitted to be less than, equal to, or even greater than the effect 10 mg everolimus. For the vast majority of patients, the model estimated a 10-mg dose of everolimus to be more effective than a 5-mg dose at shrinking their SLD. Our model assessed the effect of dose on growth of target lesions; thus, it did not capture any potential benefit that a reduced dose of everolimus may have on nontarget lesions and/or the prevention of new lesions. Subsequent efforts to model the dose–response of nontarget and new lesions demonstrated a marked difference between placebo and a 10-mg dose of everolimus, but no difference between 5-mg and 10-mg doses of everolimus was detected [22]. It may be that there is no difference between the two everolimus doses on nontarget and new lesions or that the lack of difference may have been because the measurements of these lesion types were categorical rather than continuous; characterization of the dose–response relationship for these variables requires additional data.

In a typical statistical analysis of a clinical trial, standard techniques such as the log-rank test are performed to compare outcomes in different treatment arms [23]. These techniques have the advantages of simplicity and a long history of use, but a disadvantage is that information about individual patients is ignored. The nonlinear mixed-effects modeling approach presented here can be used to describe not only the overall population, but also the tumor dynamics of each individual patient. This method offers the possibility to predict clinical observations as a function of time, and enables evaluation of the impact of actual dosing history and individual covariates on the drug effect observed. Modeling a continuous variable (tumor size) has enabled us to quantify a difference in the effect of 10 mg versus 5 mg everolimus, a difference that a coarser approach based on modeling categorical variables, such as RECIST response or PFS, might have missed.

In recent years, an increasing number of reports on modeling tumor dynamics have appeared in the literature and a variety of models have been employed that vary in the following three ways. 1) For placebo-treated patients, tumor growth has been described as linear, exponential, logistic, and/or gompertzian [19-21,24]. For the placebo growth model, we note that therapy is generally changed once a patient progresses; thus, the long-term steady state in the gompertz and logistic equations is rarely observed (Figure 1). 2) Furthermore, the drug effect can affect either the growth or decay term in the equation. In our case, we have tried both functional forms, but since they have similar analytic solutions, both describe our data reasonably well. 3) Finally, one can introduce a delay in the time it takes for a drug to affect the tumor [19]. We found that adding delay between the dose and the drug effect did not significantly improve the model fits, and we note that the time-scale for tumor shrinkage is on the order of months, whereas the terminal half-life of everolimus when dosed at 10 mg daily is around 30 h [9], so any delay is likely short compared with the time scales of interest in the study. In future work, we plan to formally compare these different models. It should be noted that multiple models would likely be suitable to describe the data presented herein.

Tumor growth in the majority of RECORD-1 patients was well described by our model, with exceptions observed in <2% of patients. Of these patients (*n* = 7), 4 displayed initial shrinkage followed by growth over the course of treatment. Because of the small sample size, further modeling of the tumor dynamics in these particular patients was not conducted.

Simulations of tumor size in patients after 1 year of continuous treatment with everolimus show that a significant antitumor effect is achieved with either a 5-mg or 10-mg daily dose, but that a substantially improved response (tumor shrinkage) can be expected in patients receiving the 10-mg daily dose. These results support earlier clinical studies that identified 10 mg daily as the preferred clinical dose based on the complete inhibition of mTOR pathway signaling observed in tumor tissue from patients receiving this dosing regimen [8,9,11]. While our results suggest that, whenever possible, clinical dosing of everolimus should be maintained at 10 mg daily, the model also demonstrates that a reduction in tumor burden compared with placebo can be achieved even in patients who require a dose reduction to 5 mg daily. This observation is noteworthy, as dose reductions to 5 mg daily are an integral part of the clinical strategy recommended by a panel of RECORD-1 investigators for the management of noninfectious pneumonitis (grade 2/3), infection (grade 2/3), stomatitis (grade 3), and metabolic abnormalities (grade 3) that arise as a result of everolimus therapy [25].

To definitively show that a 10-mg dose of everolimus is superior to a 5-mg dose, a clinical trial would be required with 10 mg everolimus and 5 mg everolimus arms that is powered to measure an outcome difference (in PFS or overall survival [OS]) between the two doses. The RECORD-1 trial was not designed to compare these two different treatment arms (5 mg and 10 mg) to placebo, and such a 3-arm trial would have required more patients. Even if such data were available, one would need to consider that dose in the 10 mg everolimus arm might have been reduced in some patients due to AEs. The present analysis allows for the detection of a difference in the target lesion response to the 2 different doses in RECORD-1 by modeling the relationship between the dose given over time and tumor size. Thus, this work complements the initial biomarker analysis [11] demonstrating that not only is a 10-mg daily dose of everolimus more effective than a 5-mg daily dose at reducing downstream mTOR signaling, but also that a 10-mg daily dose is more effective than a 5-mg daily dose at shrinking target lesions.

## Conclusions

In summary, a pharmacodynamic model of tumor response has been developed that utilizes the everolimus dosing history and tumor time course of each patient from the RECORD-1 trial to directly link everolimus dose to tumor size. Our analysis demonstrates that a daily dose of 10 mg is more efficacious than a daily dose of 5 mg at reducing tumor growth in patients with mRCC, and supports earlier studies that established 10 mg daily as the preferred clinical dose of everolimus. These results have direct implications for patients currently receiving everolimus therapy for whom dose modification may be an appropriate treatment strategy. Since tumor size is used directly in the calculation of PFS, the primary trial end point, this model may provide improved understanding of the everolimus dose–response relationship relative to methods that utilize measures of mTOR pathway inhibition.

## Methods

### Patients

Study design for the double-blind, randomized, phase 3 RECORD-1 study (Clinicaltrials.gov identifier: NCT00410124) has been described previously [13,14]. Patients with clear cell mRCC who had progressed on, or who were intolerant of, treatment with sunitinib or sorafenib were enrolled. Prior therapy with cytokines and/or VEGF inhibitors (e.g., bevacizumab) was permitted. Patients were randomized (2:1) to receive either everolimus 10 mg daily (*n* = 277) or placebo (*n* = 139) plus best supportive care. Dose reduction to 5 mg everolimus and/or treatment interruption was allowed for toxicity.

Disease progression was defined according to RECIST [15]; at least a 20% increase in the SLD of all target lesions compared with the smallest (nadir) SLD of all target lesions recorded at or after baseline and/or occurrence of a new lesion and/or unequivocal progression of existing nontarget lesions.

The protocol was approved by the institutional review boards of the participating institutions and the study was done in accordance with international standards of good clinical practice. All patients provided written informed consent. The study was conducted according to the ethical principles of the Declaration of Helsinki.

### Pharmacodynamic data collection and analysis

All RECORD-1 patients with at least one baseline tumor measurement were included in this retrospective analysis. The dataset employed included SLD data collected through the final cut-off date of February 28, 2008. Tumor measurements (performed by CT or MRI scan) were taken at screening and every 8 weeks for the remainder of the study. Target lesions were identified at baseline per RECIST criteria; ≤5 measurable lesions per organ and 10 lesions in total, representative of all involved organs [15]. Target lesions were selected based on size (longest diameter) and suitability for accurate repeated measurements (RECIST) [15]. Most target lesions were metastatic, most frequently appearing in the lung, liver, and lymph nodes. SLD for all target lesions was calculated and reported as baseline SLD. Selection of target lesions and tumor assessments were conducted both by local investigators and by blinded independent central review. However, patients initially randomized to the placebo arm who crossed over to open-label everolimus following disease progression were subsequently followed by local investigators only. For this reason, the local investigator-assessed dataset was employed in our analysis.

Nonlinear mixed-effects modeling was conducted using the First Order Conditional Estimation (FOCE) method with the $PRED in NONMEM (version VI, GloboMax LLC). The modeling utilized measurements of SLD at various time points and was defined for patient *i* by the equation *dy*_*i*_/*dt* = *r*_*i*_ – *E*_*dose,i*_*y*_*i*_, in which *y* = sum of the longest tumor diameters, *dy*/*dt* = rate of change of tumor size, *r* = net tumor growth rate for placebo-treated tumors, *E*_*dose*_ = effect of everolimus on tumor growth as a function of the daily dose. An additive error *ϵ* was then added to y(t). The model parameters (*r*_*i*_, *E*_*10,i*,_*E*_*5,i*_) were assumed to be normally distributed. Using the equation above, 2 different dose-effect models for *E*_*dose*_ were explored. The initial tumor size (*y*_0_) was set to the observed baseline tumor assessment of each patient. Alternatively, we could have treated y_0_ as a free parameter that is fit to the data. We chose this approach instead because we found that the observed y_0_ did not follow a true log-normal distribution, and forcing this distribution via a model led to poor fits for patients with very large or very small initial SLDs. We note that this framework allows for daily dose changes to directly affect the target lesion dynamics.

In model 1, it was assumed that the effect of 5 mg would be equal to the effect of 10 mg. In model 2, it was assumed that the effect of 5 mg and 10 mg would be different [*E*_*5*_ *≠ E*_*10*_].

(2) $$\begin{array}{l} \text{Model }1:E_{dose,i}=\left\{ \begin{array}{c} E_{10,i}\text{ when dose}>0\\ 0\text{ when dose}=0 \end{array}\right.\\ \text{Model }2:E_{dose,i}=\left\{ \begin{array}{c} E_{10,i}\text{ when dose}=10\;\text{mg}\\ E_{5,i}\text{ when dose}=5\;\text{mg}\\ 0,\text{ when dose}=0\; \end{array}\right. \end{array}$$

The possibility that there was a covariance relationship between the model parameters was also tested.

In this model, the net growth rate of the tumor (*dy/dt*) was defined as the change in tumor size over time, which may depend upon a number of factors, including cell proliferation, apoptosis, necrosis, and change in size of individual tumor cells. The biological mechanisms underlying change in tumor size cannot be determined based on SLD alone; thus, the model is agnostic as to the specific mechanisms governing SLD growth dynamics.

### Prognostic factor data collection and analysis

A number of prognostic factors for PFS and OS in the RECORD-1 trial have been previously reported [14]. We explored the effect of a subset of these factors on SLD dynamics, including: hemoglobin, baseline Karnofsky performance score, corrected serum calcium, number of other organs involved (including liver, bone, lymph nodes, and central nervous system), prior therapy (with either sunitinib or interferon), neutrophil count, and alkaline phosphatase. In addition, baseline SLD was included as a variable based on results from an exploratory analysis. In a process called forward-inclusion [26], each factor was added in a step-by-step manner to the base model described above, and the improvement in model fit was assessed using a likelihood ratio test. If the factor that led to the largest improvement in fit was found to have a statistically significant improvement (*P* < 0.05), then that factor was added to the model. Using the resulting new model, the process was repeated until no additional factors of significance were identified. In a subsequent process (backward-exclusion [26]), each factor was then removed step-by-step, and the original and new models assessed. If the model containing more factors had an improved fit that was highly significant (*P* < 0.001), then the factor was retained in the final model. In addition, to ensure that the prognostic factor not only improved the likelihood of the model but also improved the explanatory power of the model, we also required the factor to reduce the intersubject variability of the model parameters (η_r_ η_E10_).

Each prognostic factor or covariate was assumed to have a multiplicative effect on the parameter of interest, and continuous and binary covariates were treated slightly differently. To illustrate how a covariate was included, we show how the placebo growth rate *r*_*i*_ would vary with baseline SLD (*y*_*0*_, continuous) and prior sunitinib therapy (SUN, binary): for the continuous covariate, we have *r*_*i*_ = *r* × [*y*_*0*i_/*y*_*0*_]^*θ*^ + *N*(0,*η*_*r*_), where *r* is the median placebo growth rate for the population, *y*_*0i*_ is the baseline SLD for patient *i*, *y*_*0*_ is the median baseline SLD, *θ* is the strength of the effect of baseline SLD on the placebo growth rate, and *η*_*r*_ denotes the intersubject variability of the population. For binary covariates, we used a similar functional form with *r*_*i*_ = *r* × *θ*^SUN^ + *N*(0,*η*_*r*_). Here, SUN = 1 for patients with prior sunitinib therapy and SUN = 0 otherwise.

### Model qualification and simulation

Model qualification was performed by comparing clinically measured SLD with those predicted by the model, evaluating the uncertainty in parameter values, and using the model to perform a simulation of two sets of 10,000 virtual patients. For the latter, model parameters were chosen from the parameter distribution obtained in the final model, and the two sets of virtual patients simulated were: A) patients on the placebo arm before cross-over; and B) patients who maintained constant everolimus dosing throughout the study.

Model simulations of 100,000 patients were performed to estimate the mean and variability of the predicted response of patients who received placebo and for patients who received everolimus 5 mg and 10 mg continuous dosing. For each virtual patient, the fixed effects were sampled from a multivariate normal distribution based on the uncertainty of the fixed effect estimate; the random effects were sampled from a multivariate normal distribution to account for intersubject variability.

## Abbreviations

4EBP1: eukaryotic initiation factor 4E binding protein 1; AE: adverse event; CR: complete response; eIF-4E: eukaryotic initiation factor 4E; FOCE: first order conditional estimation; mRCC: metastatic renal cell carcinoma; mTOR: mammalian target of rapamycin; OS: overall survival; PBMC: peripheral blood mononuclear cells; PD: progressive disease; PFS: progression-free survival; PR: partial response; S6K1: S6 kinase 1 ribosomal protein; SD: stable disease; SLD: sum of the longest tumor diameters; VEGFr-TKI: vascular endothelial growth factor receptor-tyrosine kinase inhibitor; VPC: visual predictive check.

## Competing interests

AS, WW, OC, NH, and CS are employees of Novartis Pharmaceuticals Corporation, and AC and HK are former employees of Novartis. RM has acted as a consultant to Aveo and GlaxoSmithKline, has received honoraria from Wyeth and Novartis, and has received research funding from Pfizer, Novartis, and GlaxoSmithKline.

## Authors’ contributions

AS, WW, AC, OC, NH, and CS participated in the conception, design and analysis of the pharmacodynamic model reported herein. RM was the Principal Investigator of the RECORD-1 study and participated in its design and in data acquisition. All authors were involved in the drafting of the manuscript, and all authors consented to its submission for publication.

## Authors’ information

Alison A. Carter and Hyewon Kim are former employees of Novartis.

## Pre-publication history

The pre-publication history for this paper can be accessed here:

http://www.biomedcentral.com/1471-2407/12/311/prepub

## Supplementary Material

Additional 1 **Fig. A1 **Tumor growth in a small number of RECORD-1 patients was not well described by model 2 with E5 ≠ E10. These included: i-iv) resistant patients (n = 4); (v) 5 mg of everolimus had an equivalent effect to 10 mg of everolimus (n = 1); (vi) tumor shrinkage during placebo treatment, but progression due to nontarget lesions (n = 1); (vii) tumor growth at 10 mg everolimus is faster than tumor growth at 5 mg everolimus (n = 1). E5, treatment effect of everolimus 5 mg daily; E10, treatment effect of everolimus 10 mg daily; SLD, sum of the longest tumor diameters. Click here for file
