# Supplementary material for: Dynamic tumor modeling of the dose–response relationship for everolimus in metastatic renal cell carcinoma using data from the phase 3 RECORD-1 trial
Source: BMC Cancer. 2012 Jul 23;12:311. doi: 10.1186/1471-2407-12-311 (PMC3495014; doi:10.1186/1471-2407-12-311)
Supplement: Additional 1 — Fig. A1 Tumor growth in a small number of RECORD-1 patients was not well described by model 2 with E5 ≠ E10. These included: i-iv) resistant patients (n = 4); (v) 5 mg of everolimus had an equivalent effect to 10 mg of everolimus (n = 1); (vi) tumor shrinkage during placebo treatment, but progression due to nontarget lesions (n = 1); (vii) tumor growth at 10 mg everolimus is faster than tumor growth at 5 mg everolimus (n = 1). E5, treatment effect of everolimus 5 mg daily; E10, treatment effect of everolimus 10 mg daily; SLD, sum of the longest tumor diameters. [file 1471-2407-12-311-S1.doc]

**APPENDIX**

**Fig. A1.** Tumor growth in a small number of RECORD-1 patients was not well described by model 2 with E_5_ ≠ E_10_. These included: i-iv) resistant patients (*n* = 4); (v) 5 mg of everolimus had an equivalent effect to 10 mg of everolimus (*n* = 1); (vi) tumor shrinkage during placebo treatment, but progression due to nontarget lesions (*n* = 1); (vii) tumor growth at 10 mg everolimus is faster than tumor growth at 5 mg everolimus (*n* = 1). E_5_, treatment effect of everolimus 5 mg daily; E_10_, treatment effect of everolimus 10 mg daily; SLD, sum of the longest tumor diameters.

**
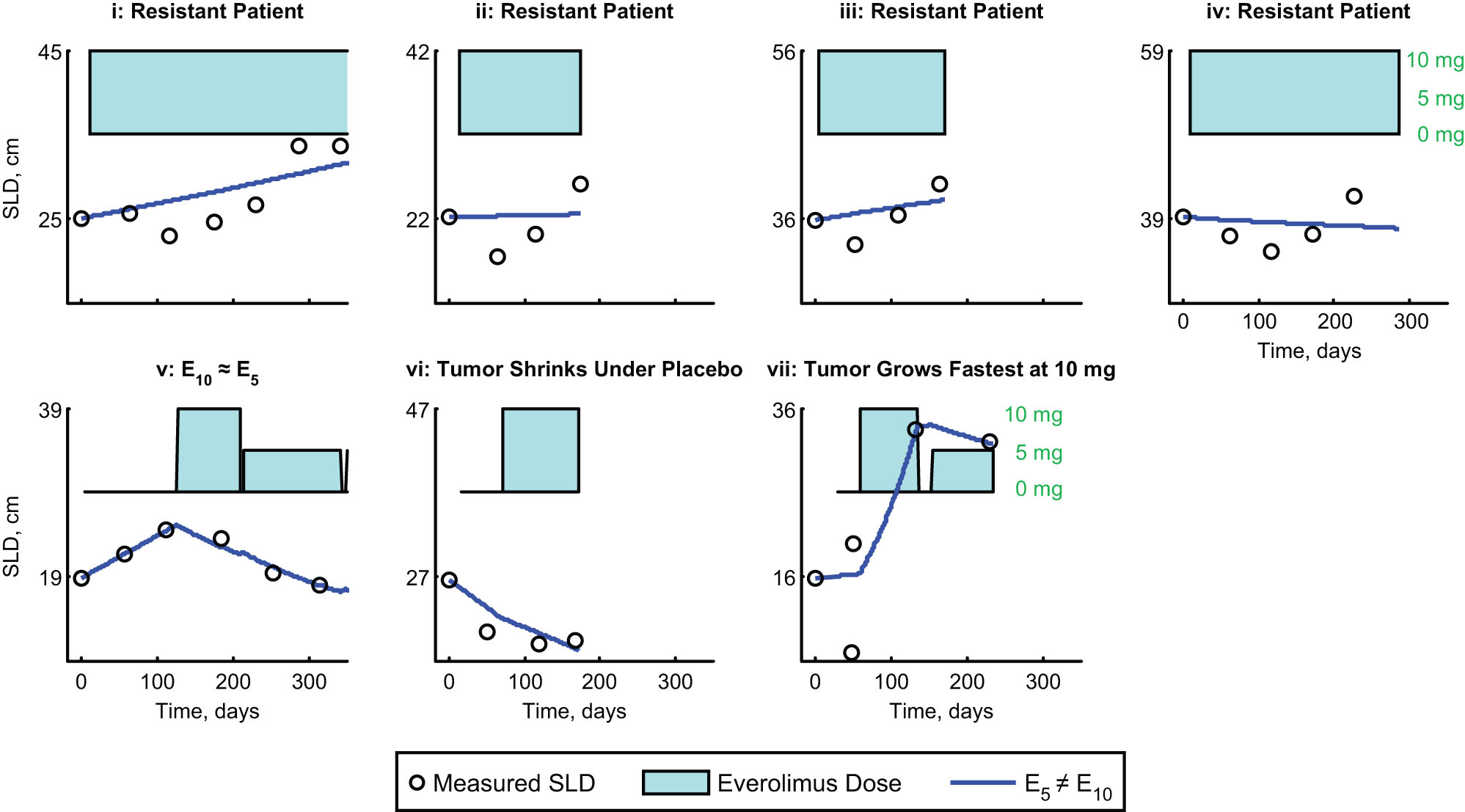
**
